# Endobronchial Tuberculosis: Two Case Reports and Review of the Literature

**DOI:** 10.1155/2014/283972

**Published:** 2014-08-18

**Authors:** Anshum Aneja, Uma Maheswari Krishnaswamy, Vijayashree Thyagaraj, Riyaz P. Moideen, Mantha Satya Padmaja

**Affiliations:** ^1^Department of Respiratory Medicine, M.S. Ramaiah Medical College, MSRIT Post, Bangalore, Karnataka 560054, India; ^2^Department of Internal Medicine, M.S. Ramaiah Medical College, MSRIT Post, Bangalore, Karnataka 560054, India

## Abstract

Endobronchial tuberculosis commonly affects young patients and presents as acute or insidious onset cough, wheeze, low grade fever, and constitutional symptoms. Although endobronchial lesions usually result in sputum positivity for acid fast bacilli (AFB), a false negative sputum or absence of radiological lesions may result in delayed diagnosis. On the other hand, sputum positivity with presence of signs on chest radiology may lead to consideration of parenchymal TB as the primary diagnosis and the coexistence of endobronchial lesions may be missed until sequelae of the latter ensue. Besides, in elderly patients, consideration of other differentials like malignancy and pneumonia may lead to misdiagnosis. Hence, bronchoscopy is essential for confirmation of endobronchial TB. We hereby report two cases of endobronchial TB which stress the importance of bronchoscopic diagnosis for timely institution of treatment and prevention of permanent sequelae, respectively.

## 1. Introduction

Endobronchial tuberculosis (TB) occurs in about 10–40% of patients with active tuberculosis [1]. More than half the cases of endobronchial TB occur in patients aged less than 35 years old [2]. The common symptoms of endobronchial TB include cough with expectoration, hemoptysis, breathlessness, and wheeze [3]. The occurrence of an irritable barking cough unresponsive to antitussive medication has also been described as a clinical presentation of endobronchial TB [4]. However, this entity remains a diagnostic challenge even in countries with a high prevalence of TB.

Despite widely available diagnostic testing, endobronchial TB is a major cause of morbidity as it frequently heals with concentric scarring resulting in bronchostenosis and atelectasis [4]. Sputum examination is the first step towards the diagnosis of endobronchial TB. However, in cases where sputum is negative or chest radiographic findings are equivocal, bronchoscopy and computed tomography are the investigative modalities of choice to detect and plan appropriate treatment. The commonly reported bronchoscopic findings include hypertrophy with luminal narrowing, mucosal edema, erosion, ulceration, and cicatricial stenosis with pseudomembrane formation [5].

We report two cases in this series, wherein the importance of bronchoscopy as a tool in the timely diagnosis and recognition of sequelae of endobronchial TB has been highlighted.

## 2. Case 1

A previously healthy 65-year-old female patient was admitted in the medical unit of our hospital with a history of fever and persistent cough 1 month ago. General examination was unremarkable except for the presence of pallor. Respiratory system examination revealed bronchial breath sounds and crackles in the right infraclavicular area. Examination of other systems was normal. A panel of laboratory tests was performed which confirmed iron deficiency anemia.

**Figure 1:**
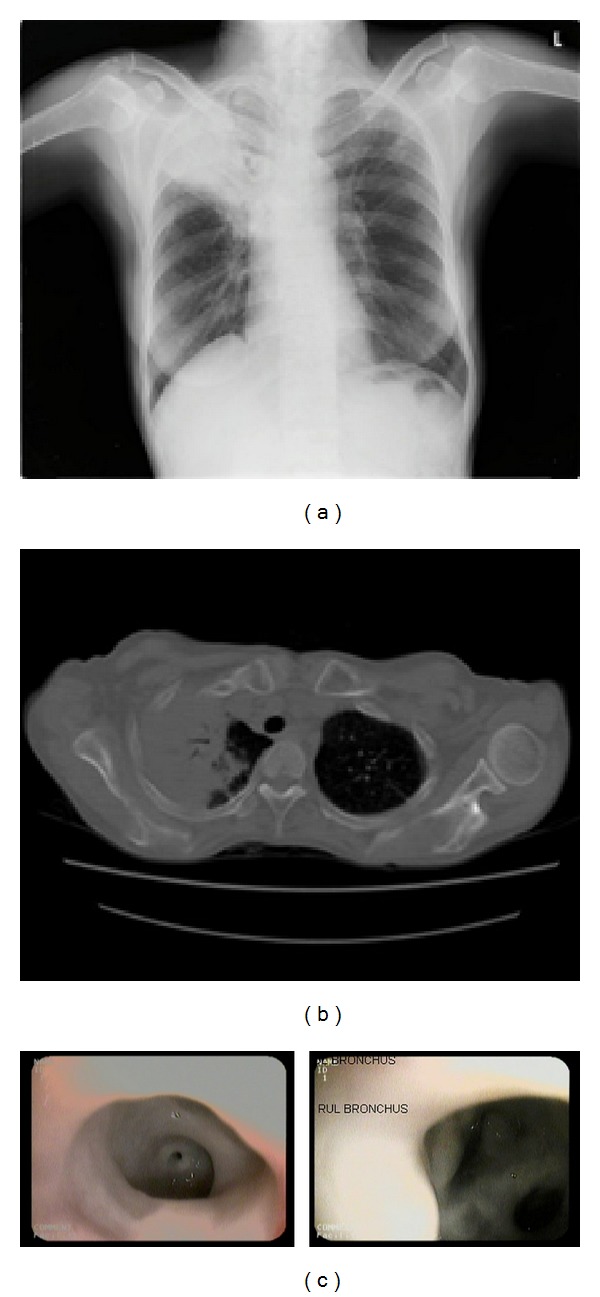
(a) Chest X ray at presentation showing right upper lobe homogenous opacity. (b) CT chest showing dense peripheral consolidation of the right upper lobe. (c) Bronchoscopic picture of right upper lobe showing cheesy pseudomembrane lining the right upper lobe bronchial segments.

Chest radiogram showed right upper zone homogenous opacity. The patient was diagnosed to have community acquired pneumonia and was treated with broad spectrum antibiotics. She was referred to pulmonary medicine in the second week of admission to rule out post-obstructive pneumonia, as there was no clinical response to the above mentioned treatment. A computed tomography (CT) of the thorax was performed; it revealed dense peripheral consolidation of the right upper lobe with enlarged pre- and paratracheal lymph nodes (Figures 1(a) and 1(b)). As the lesion was deemed not amenable for CT guided biopsy, bronchoscopy was done; it showed mucoid, tenacious, and thick cheese-like pseudomembrane completely lining the right upper lobe bronchial subsegments with a narrow but patent bronchial lumen (Figure 1(c)). All other bronchial segments were normal. Bronchial washing from the right upper lobe bronchus was positive (+++) for acid fast bacilli (AFB). Biopsy of the pseudomembrane-like lining was attempted; satisfactory samples could be obtained as the latter was very tenacious. A diagnosis of endobronchial and parenchymal TB was made and antitubercular therapy was instituted.

## 3. Case 2

A 26-year-old female was referred to us for evaluation of severe left sided chest pain of 1-month duration. A review of her records revealed that she had received directly observed therapy (WHO Category 1) for sputum positive pulmonary TB and had been declared cured 6 months prior to presentation. On examination, general physical examination was normal. Respiratory system examination revealed volume loss and absent breath sounds over left hemithorax suggestive of left lung collapse/fibrothorax. Sputum for AFB was negative.

**Figure 2:**
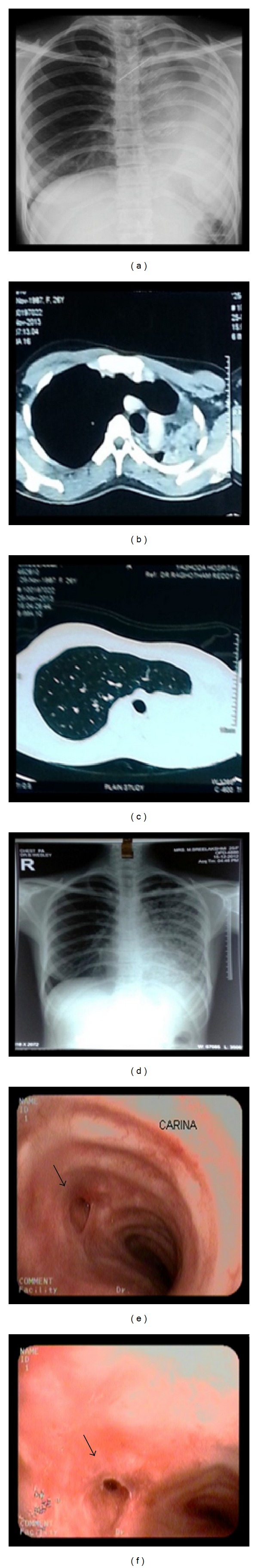
(a), (b), and (c) Chest X ray and CT at presentation showing left lung collapse with mediastinal shift and compensatory hyperinflation of the right lung. (d) Pretreatment chest X ray showing extensive left sided miliary nodules. (e) and (f) Bronchoscopic appearance of pinhole sized left main bronchial opening (arrows).

Chest radiogram showed complete collapse of left lung with ipsilateral mediastinal shift (Figure 2(a)). CT of the thorax confirmed the chest radiographic findings (Figures 2(b) and 2(c)). A review of the patient's pretreatment chest X ray showed presence of left sided miliary shadows but no evidence of lung collapse (Figure 2(d)).

In view of lung collapse occurring after successful treatment of TB, bronchoscopy was undertaken to rule out endobronchial obstruction. Bronchoscopy showed complete occlusion of left main bronchus with only a pinhole sized opening (Figures 2(e) and 2(f)). Bronchoalveolar lavage was negative for AFB on staining and on culture. Endobronchial biopsy showed chronic inflammation with no granulomas. As the left main bronchus was completely cicatrized, the patient was unsuitable for stenting. A diagnosis of left main bronchostenosis secondary to tuberculosis was made and the patient was referred to the cardiothoracic surgeon for bronchial reconstruction/bronchoplasty. However, the patient declined to undergo any surgical procedure.

## 4. Discussion

Endobronchial TB is defined as tuberculous infection of the tracheobronchial tree. Endobronchial TB is commoner in young adults and exhibits a female preponderance [6]. However, about 15% of geriatric patients may also have endobronchial TB [7]. The common symptoms of endobronchial TB include cough with expectoration, hemoptysis, breathlessness, and wheeze. Since TB is also a common cause of nonresolving consolidations and fever of unknown origin, endobronchial spread of the disease may lead to unexpected outcomes and poor response. The most common sites involved in endobronchial tuberculosis are right upper lobe and right main bronchus. The roentgenographic appearances involve consolidation or loss of volume. However, a normal chest roentgenogram does not exclude endobronchial pathology.

It is known that the key to the diagnosis of endobronchial TB is a high index of suspicion and prompt performance of diagnostic bronchoscopy. Apart from visualization of bronchial tree abnormalities suggestive of endobronchial TB, fibreoptic bronchoscopy can also provide excellent material for diagnosis of suspected cases of pulmonary TB especially when sputum smears are negative for AFB [8].

In our first patient, an elderly lady with nutritional anemia, the presence of an endobronchial component was diagnosed early. She was referred to us in view of a nonresponse to standard of care for community acquired pneumonia. However, the second patient was a young lady with a late presentation of endobronchial TB, who had been successfully treated for sputum positive parenchymal TB with a neglected endobronchial component. She presented with symptoms attributable to progressive fibrostenosis. The endobronchial component might have been missed in this case as the patient had been treated under programmatic conditions wherein a positive sputum AFB is considered as definitive proof of parenchymal TB and the patient is not followed up after microbiological cure.

Endobronchial TB is divided into seven subtypes based on bronchoscopic appearance, namely, (i) actively caseating, (ii) edematous-hyperemic, (iii) fibrostenotic, (iv) tumorous, (v) granular, (vi) ulcerative, and (vii) nonspecific bronchitis [9]. Our first case fulfilled the diagnostic appearance of actively caseating type of endobronchial TB in view of a diffuse cheesy pseudomembrane. While the actively caseating form is the most common subtype, its prognosis is usually grave, resulting in fibrostenosis in two-thirds of patients [10].

The first patient in our report had actively caseating type of endobronchial TB of the right upper lobe with a patent bronchial lumen. However, the fibrostenotic component in the second patient represented a sequel of an overlooked circumferential involvement of the left main bronchus which appeared as the classically described “crushed waterdrop.”

The prognosis of fibrostenotic endobronchial TB is poor. In the original series by Bachh et al. [8], all cases described remained in a fibrostenotic state during treatment and nearly half of these patients showed complete bronchial obstruction even after 2 to 3 months after treatment. Hence, it may be useful to follow up patients with unilateral alveolonodular or miliary infiltrates to rule out and minimize stenotic outcomes due to coexistent endobronchial TB. Though bronchial stenosis is an irreversible delayed complication of endobronchial TB, systemic corticosteroids, balloon dilatation, endobronchial stenting, and surgical interventions should be considered as an add-on to standard antituberculosis therapy [11, 12].

In conclusion, in TB endemic countries, patients with unilateral infiltrates or sputum negativity with suggestive symptoms must be evaluated bronchoscopically for microbiological diagnosis and to rule out endobronchial TB.
